# Fausse couche de 8 semaines d´aménorrhée chez une patiente positive au virus SARS-CoV-2

**DOI:** 10.11604/pamj.supp.2020.37.1.23445

**Published:** 2020-10-22

**Authors:** Mohammed Karam Saoud, Salma Lamsyah, Nissrine Mamouni, Sanaa Errarhay, Chahrazad Bouchikhi, Abdelaziz Banani

**Affiliations:** 1 Département de Gynécologie-Obstétrique, Centre Hospitalier Universitaire Hassan II, Fès, Maroc

**Keywords:** SARS-CoV-2, grossesse, fausse couche, SARS-CoV-2, pregnancy, miscarriage

## Abstract

Un nouveau coronavirus (SARS-CoV-2) mis en évidence en fin d´année 2019 en Chine se diffuse à travers le monde. Le plus souvent à l´origine d´un syndrome infectieux sans gravité, associant à différents degrés des symptômes bénins (fièvre, toux, myalgies, céphalées et éventuels troubles digestifs). Le SARS-Cov-2 peut être à l´origine de pathologies pulmonaires graves et parfois de décès. Nous présentons ici un cas d´une patiente âgée de 21 ans qui consulte pour une fausse couche de 8 semaines d’aménorrhée (SA) et chez qui on a diagnostiqué une infection concomitante par le virus SARS-CoV-2. L´intérêt de cette observation est de faire un rappel sur le mode de transmission, de discuter la gravité du virus en cas de grossesse et d´étaler le protocole thérapeutique utilisé dans notre pays devant cette épidémie.

## Abstract (en)

A new coronavirus (SARS-CoV-2) highlighted at the end of 2019 in China is spreading around the world. Most often the cause of an infectious syndrome, associating mild symptoms (fever, cough, myalgia, headache and possible digestive disorders) to different degrees. SARS-CoV-2 can also cause serious lung disease and sometimes death. Here we present a case of a 21-year-old woman who consults for a miscarriage of 8 weeks of amenorrhea and has been diagnosed with concomitant SARS-CoV-2 infection. The importance of this observation is to make a remind of the mode of transmission, to discuss the severity of the virus in case of pregnancy and to discuss the therapeutic protocol used in our country in front of this epidemic.

## Introduction

L´infection au coronavirus (COVID-19) représente une urgence sanitaire mondiale. Au moment de la rédaction de l´article, la COVID-19 a touché plus de 2 millions de personnes au monde, occasionnant 284 000 morts et 1 million 500 milles guérisons. Peu d´études ont été réalisées sur la population des femmes enceintes, malgré que cette population doive requérir une attention particulière du fait que la grossesse représente un état physiologique qui rend les femmes vulnérables aux infections virales. Nous présentons ici le cas d´une femme qui présente une fausse couche de 8 SA chez qui une infection à la COVID-19 fut découverte.

## Patient et observation

Il s´agit d´une patiente âgée de 21 ans, sans antécédents pathologiques notables, sans notion de contact avec une personne COVID+, G3P1, qui consulte pour prise en charge de métrorragies sur une aménorrhée de 8 semaines. L´examen clinique trouve une patiente consciente stable sur le plan hémodynamique et respiratoire, au spéculum un saignement endo-utérin et au toucher vaginal un col ouvert à 1 doigt large. Une échographie endovaginale réalisée objectivant un sac gestationnel avec une échographie embryonnaire à activité cardiaque négative, isthmique, en voie d´expulsion. La décision était de mettre la patiente sous syntocinon avec une surveillance jusqu´à l´expulsion. Deux heures après, la patiente a expulsé le produit de conception, déclarée sortante avec échographie de contrôle prévue dans une semaine. Trois jours plus tard, la patiente reconsulte pour une fièvre, une toux et des métrorragies minimes, l´examen clinique trouve une patiente fébrile à 39°, la fréquence respiratoire à 24 cycles/min, la saturation à 97% à l´air ambiant, au spéculum métrorragies minimes provenant de l´endocol sans leucorrhée ni douleur à la mobilisation de l´utérus, à l´échographie on note la présence d´une image de rétention de 15mm. Le diagnostic initial retenu était une endométrite, la patiente a été mise sous amoxicilline protégée et métronidazole.

**Figure 1:**
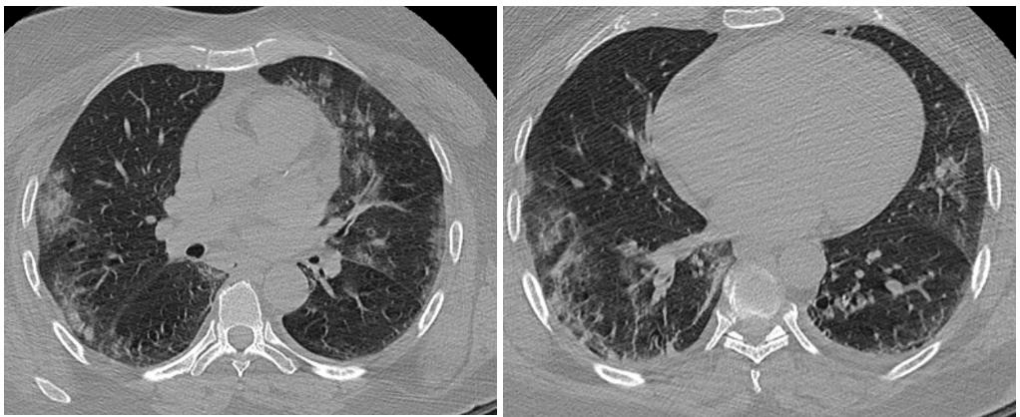
opacité en verre dépoli, bilatérale, diffuse, périphérique et centrale, lobaire supérieur, moyen et inférieur avec foyers de condensation bilatéraux périphériques, lobaire supérieur et inférieur

Vu la non amélioration de la symptomatologie et vu la situation endémique de la COVID-19, un test PCR sur prélèvement naso-pharyngé à la recherche du virus SARS-CoV-2 a été réalisé, dont le résultat est revenu positif confirmé par un deuxième test. La patiente fut isolée, hospitalisée dans un service dédié à la COVID-19, mise sous le protocole thérapeutique conseillé par le Ministère de la Santé du Maroc à base de chloroquine (Nivaquine) 500 x 2 pendant 10 jours-Azithromycine 500mg à J1 puis 250 mg de J2 à J7, héparine à bas poids moléculaire à dose préventive et du paracétamol. Un bilan biologique réalisé, revenu normal à part une protéine C réactive (CRP) à 100 contrôlée chaque 48h évoluant de 76 à 52 avant de se normaliser le 10^e^ jour. Un scanner thoracique réalisé objectivant des opacités en verre dépoli bilatérales associées à des foyers de condensation périphériques bilatéraux (Figure 1). La patiente a bénéficié d´une surveillance quotidienne de la température qui s´est normalisée le 2^e^ jour, des signes fonctionnels respiratoires avec apparition d´une légère dyspnée le 5^e^ jour d´hospitalisation et d´une surveillance des effets secondaires de la chloroquine notamment les effets secondaires cardiaques. Une amélioration clinique obtenue le 10^e^ jour avec disparition de la dyspnée. La patiente a été déclarée sortante le 14^e^ jour après réalisation de deux tests PCR espacés de 3 jours revenant négatifs. Un scanner thoracique de contrôle est prévu dans 3 mois.

## Discussion

La transmission interhumaine du virus SARS-CoV-2 a été démontrée en février 2020 après qu´une contamination intrafamiliale ait été rapportée [1]. La principale voie de transmission interhumaine est la voie aéroportée (gouttelettes ou aérosols), par ailleurs, la présence d´ARN viral dans le sang ou les selles a fait évoquer les possibilités d´une contamination sanguine ou oro-fécale, qui n´ont toutefois pas été démontrées à ce jour [2]. Dans l´étude de Guan *et al*. portant sur 1099 patients vus rétrospectivement, la durée médiane d´incubation et la date d´apparition des symptômes, était estimée à 4 jours [3]. D´autres études ont estimé cette durée à 5,1 et 5,2 jours [4,5]. La contagiosité du virus est plus importante lors des premiers jours de symptômes et qu´elle pourrait persister plus de trois semaines. La majorité des personnes (80%) qui ont été infectées par le SARS-CoV-2 n´ont eu que de légers symptômes en particulier une toux, une fièvre et une dyspnée. Cependant, des symptômes plus graves ont également été décrits dans ce contexte (16 à 32%) comme la pneumonie ou le syndrome de détresse respiratoire aiguë (SDRA) [6]. Les changements physiologiques maternels normaux accompagnant la grossesse avec une modification de l´immunité et des échanges cardiopulmonaires pourraient être à l´origine de la plus grande sensibilité et de l´augmentation de la gravité clinique de la pneumopathie. Concernant le SARS-CoV-2, la revue de la littérature actuelle, bien que très limitée chez la femme enceinte, semble montrer que les symptômes sont les mêmes que ceux de la population générale pour la grande majorité des femmes qui donc ne ressentiraient que de légers symptômes de rhinite ou un syndrome grippal avec potentiellement de la toux, une fièvre ou une dyspnée. Mais ces femmes peuvent également présenter des symptômes plus graves tels que la pneumonie ou le SDRA comme les autres populations à risque [7].

Les données chez la femme enceinte pour le SARS-CoV-2 étant très limitées, des rapprochements peuvent être faits avec ce qui est connu dans le cadre des autres pneumopathies ou des autres coronavirus tels que le SARS-CoV ou le MERS-CoV. Les patientes avec des pneumopathies seraient plus à risque de rupture prématurée des membranes, d´accouchements prématurés, de morts fœtales in utero, de retards de croissance intra-utérins et de décès néonataux [8]. Il y avait également dans certains cas des issues obstétricales défavorables avec des fausses couches, des accouchements prématurés et des décès maternels [9]. Les formes symptomatiques de l´infection à SARS-CoV-2 s´accompagnent des modifications biologiques suivantes [10]: élévation des polynucléaires neutrophiles et lymphopénie, élévation de la CRP jusqu´à 150 mg/L, hypoalbuminémie, hyperferritinémie. Une élévation des ALAT/ASAT dans environ 25% des cas et une hyperbilirubinémie. Une élévation des LDH pour environ 40% des patients associée à une diminution du TP jusqu´à 94% des patients et à une augmentation des D-dimères, stigmates d´une coagulopathie associée aux formes graves et prédictives de la mortalité. Une élévation de la troponine chez 17% des patients et une alcalose respiratoire chez 28% des patients secondaire à la polypnée. Concernant notre patiente le bilan biologique était normal, à part la CRP qui s´est normalisée le 10^e^ jour.

Les signes radiologiques sont peu spécifiques: dans l´étude de Guan *et al*. [3], les patients présentaient des images en verre dépoli dans 56,4% des cas, des condensations alvéolaires unilatérales dans 41,9% des cas et bilatérales dans 51,8% des cas, avec anomalies interstitielles dans 14,7% des cas. Bien que notre patiente soit paucisymptomatique, le scanner thoracique révèle des opacités en verre dépoli et de petits foyers de condensation. Des publications récentes ont attiré l'attention sur les avantages possibles de la chloroquine, utilisée principalement comme un antipaludéen, dans le traitement des patients infectés par le coronavirus (SARS-CoV-2) [11]. L'activité antivirale in vitro de la chloroquine a été identifiée depuis la fin des années 1960 [12]. Actuellement, aucune étude n´a pu prouver l´efficacité de la chloroquine dans le traitement du coronavirus SARS-CoV-2. La possibilité d'utiliser la chloroquine dans le traitement du SARS-CoV-2 doit être étudiée avec attention, ainsi l´attente des résultats définitifs pour décider de son efficacité est nécessaire. Au Maroc, cette molécule est utilisée en association avec l´azithromycine et une héparinothérapie depuis le 23 mars 2020, ainsi notre patiente a bénéficié du protocole recommandé.

## Conclusion

Peu d´études ont été menées sur la relation entre la COVID-19 et le déroulement de la grossesse, et aucune étude n´a été menée sur la relation entre l´infection par le coronavirus et les fausses couches. Mais par analogie avec le SARS ou le MERS, sur de très petites séries, il y avait dans certains cas des issues obstétricales défavorables avec des fausses couches, des accouchements prématurés et des décès maternels [9]. D´autres études, quant à elles ne montraient pas de relation significative entre l´infection et le risque de fausse couche ou de perte foetale au deuxième trimestre [13]. D´autres études avec un échantillon représentatif sont nécessaires afin de déterminer s´il existe une relation causale entre l´infection par le SARS-CoV-2 et la survenue d´une fausse couche.
